# Kicked General Fractional Lorenz-Type Equations: Exact Solutions and Multi-Dimensional Discrete Maps

**DOI:** 10.3390/e27111127

**Published:** 2025-10-31

**Authors:** Vasily E. Tarasov

**Affiliations:** 1 Skobeltsyn Institute of Nuclear Physics, Lomonosov Moscow State University, Moscow 119991, Russia; tarasov@theory.sinp.msu.ru; 2 Department of Physics and Physical Chemistry, 915, Moscow Aviation Institute (National Research University), Moscow 125993, Russia

**Keywords:** fractional calculus, fractional integrals, Lorenz-type system, discrete map with memory, 26A33, 34A08, 70G60, 70Kxx

## Abstract

Lorenz-type systems are dissipative dynamical systems that are described by three nonlinear equations with derivatives of the first order and are capable of exhibiting chaotic behavior. The generalization of Lorenz-type equations by using general fractional derivatives (GFDs) and periodical kicks is proposed. GFDs allow us to use the general form of memory functions as operator kernels to describe nonlinear dynamics with memory. The exact analytical solutions of Lorenz-type equations with GFDs are derived in the general case for the wide class of nonlinearity and memory functions. Using the exact solutions, we obtain discrete maps with memory (DMMs) that describe kicked GF Lorenz-type systems with general forms of memory and nonlinearity. The proposed maps describe the exact solution of nonlinear equations with GFDs at discrete time points as the function of all past discrete moments of time. The proposed multi-dimensional DMMs are derived from kicked GF Lorenz-type equations with GFDs without any approximations. The proposed results and the method to derive multi-dimensional DMMs are derived for arbitrary dimensions. The importance and unusualness of the proposed results lies in the fact that obtained solutions for equations of the Lorenz-type system are exact analytical solutions.

## 1. Introduction

Deterministic chaos and nonlinear dynamics are actively developing fields of science [1,2,3,4,5,6]. A Lorenz system is described by three nonlinear ordinary differential equations of the first order whose behavior exhibits deterministic chaos. The Lorenz system is one of the most famous systems with strange attractors [7,8]. There are systems similar to the Lorenz system (for examples, the Rössler system [9,10]), which are called Lorenz-type systems.

Lorenz-type systems are dissipative dynamical systems that are described by three nonlinear ordinary differential equations:

(1) $$\left\{\begin{array}{cc} D_{t}^{1}x & =F_{x}\left(x,y,z\right),\\ D_{t}^{1}y & =F_{y}\left(x,y,z\right),\\ D_{t}^{1}z & =F_{z}\left(x,y,z\right), \end{array}\right.$$

where $D_{t}^{1}=d/dt$ is the first-order derivative with respect to the time variable. The dissipativity condition for system (1) has the form

(2) $$\mathrm{div}F\;=\;\frac{\partial F_{x}\left(x,y,z\right)}{\partial x}\;+\;\frac{\partial F_{y}\left(x,y,z\right)}{\partial y}\;+\;\frac{\partial F_{z}\left(x,y,z\right)}{\partial z}\;<\;0.$$

For the well-known Lorenz system, $\mathrm{div}F_{L}\;=\;-\sigma -1-\beta$ is the negative number [7]. Note that the dissipativity in (2) is a necessary but not sufficient condition for systems such as (1) to be chaotic. As a rule, equations of Lorenz-type systems do not have exact analytical solutions.

If, in Equation (1), we use the derivatives of the second order $D_{t}^{2}$ instead of the first derivatives $D_{t}^{1}$, we get the equations

(3) $$\left\{\begin{array}{cc} D_{t}^{2}x & =F_{x}\left(x,y,z\right),\\ D_{t}^{2}y & =F_{y}\left(x,y,z\right),\\ D_{t}^{2}z & =F_{z}\left(x,y,z\right), \end{array}\right.$$

System (3) is a non-Hamiltonian system [11] if the following condition is satisfied:

(4) curlF≠0.

For Lorenz-type systems, $\mathrm{curl}F_{L}\;\neq \;0$. Therefore, Lorenz-type system (3), in which first-order derivatives are replaced by second-order derivatives, is a non-Hamiltonian system. If condition (4) is satisfied for first-order Equation (1), then the system is a non-gradient system. All Lorenz-type systems which are described by Equation (1) are non-gradient systems.

Fractional Lorenz-type systems are described by the generalization of Equation (1) in which first-order derivatives $D_{t}^{1}$ are replaced by fractional derivatives [12,13,14,15,16]. For example, if we use a fractional derivative (FD) with a power-law kernel such as the Caputo FD, then the fractional Lorenz-type system is described by the equations

(5) $$\left\{\begin{array}{cc} D_{t}^{\alpha }x & =F_{x}\left(x,y,z\right),\\ D_{t}^{\alpha }y & =F_{y}\left(x,y,z\right),\\ D_{t}^{\alpha }z & =F_{z}\left(x,y,z\right), \end{array}\right.$$

where the order of the FD is α>0. In a more general case, one can consider different orders ($\alpha_{x}$, $\alpha_{y}$, and $\alpha_{z}$) of the FD in this system of equations. In general, Equations (1) and (3) are special cases of fractional Lorenz-type system (1) for α=1 and α=2, respectively.

The interest in using FC (see the classical 20th-century book [17,18,19], the modern books [20,21], and the handbooks [22,23]) is due to the fact that this type of calculus allows one to take into account the effects of nonlocality in time and memory. For example, see books about fractional dynamics [24,25], continuum mechanics [26,27,28,29], physics [30,31,32], biology [33], and economics [34]. The dynamics of systems with memory can demonstrate qualitatively new types of chaotic behavior and new types of attractors [12,15,16].

In this paper, we consider multi-dimensional nonlinear equations with general FDs and periodic kicks. For this purpose, we use the Luchko form of GFC, which was proposed in the 2021 papers [35,36,37] and then developed, for example, in [38,39,40,41,42], and its multi-kernel extension [43,44,45]. There are different types of general fractional calculus (for example, Kochubei-type GFC [46,47,48] and Hanyga-type GFC [49]). The Luchko type of fractional calculus is used in this article because it is more convenient for our purposes. In this paper, we derive exact analytical solutions of nonlinear equations with general FDs and multi-kernel general FDs. Kicked general fractional Lorenz-type equations are equations with multi-kernel general FDs and periodic kicks. These solutions are derived for the general form of nonlocality and the general form of memory functions without any approximations. Using these exact solutions, we derive general fractional Lorenz-type discrete maps with memory (GF Lorenz-type DMMs) as the exact solution at the discrete time points. To simplify consideration, we consider three-dimensional DMMs. The transformations and derivation of exact solutions and discrete maps are performed in a general form, which allows the results to be used for any multi-dimensional case.

The structure of this paper is as follows: A brief overview of the methods and results already published is given in Section 2. In Section 3, general fractional Lorenz-type systems with memory are considered. In Section 4, exact analytical solutions and discrete maps are derived for kicked GF Lorenz-type systems with memory. In Section 5, exact analytical solutions and discrete maps are derived for kicked GF Lorenz-type systems with multi-kernel memory. A brief conclusion is given in Section 6.

## 2. Brief Overview

Equations of Lorenz-type systems do not have an exact analytical solution. However, in nonlinear dynamics, there are nonlinear systems with chaotic behavior that are described by differential equations that have exact analytical solutions [1,2,3,4,5,6,24]. These nonlinear systems are described by nonlinear ordinary differential equations of integer orders with periodic kicks. These systems can be described by discrete maps that are exact solutions of differential equations at discrete moments of time. Such discrete maps include, for example, the logistic map, the universal map, the standard or Chirikov–Taylor map, the Henon map, the dissipative map, and the Zaslavsky map [1,2,3,4,5,6,24]. This approach allows us to derive the exact solutions of these nonlinear differential equations without any approximations.

Many researchers of deterministic chaos understood the importance to have a generalization of a well-known approach to solve nonlinear equations with fractional derivatives (FDs) and periodic kicks. Some scientists tried to generalize this approach to nonlinear equations with FDs to derive discrete maps with memory (DMMs) and nonlocality in time. Until 2008, nonlinear DMMs were simply postulated in some forms [50,51,52,53,54,55] that are not exact solutions of any differential or integro-differential equation. Some scientists even proposed a justification that periodic “blows” and kicks knock out the memory of nonlinear dynamic systems. From this they concluded that the discrete maps associated with equations with FDs cannot have memory. The necessity to solve the problem of finding exact solutions to nonlinear equations with FDs and periodic kicks and then deriving the corresponding DMMs was pointed out by Zaslavsky to various scientists who collaborated with him.Zaslavsky also encouraged the author of this article to solve this problem in 2006.This Zaslavsky problem was successfully solved by the author and then published in 2008 [56]. A fractional generalization of the well-known approach used for integer-order differential equations was first proposed by Tarasov and Zaslavsky in the 2008 paper [56] and then developed in subsequent works by the author. One can state that discrete maps with memory (DMMs) were first obtained from nonlinear equations with FDs without approximations [56]. Then, this approach was developed in [24,57,58] and then in the following works. Let us briefly describe all the results in this area:

- DMMs were derived from equations with Caputo and Riemann–Liouville FDs in [24,56,57,58], including the generalization of the Henon, dissipative, and Zaslavsky maps with memory in [24,59,60].
- DMMs were also derived from equations with FDs describing economic [34,61], population dynamics [62], and quantum dynamic systems [63].
- For the first time, DMMs were obtained from equations with fractional integrals (FIs) in [64].
- DMMs were obtained from equations with Erdelyi–Kober FDs in [65], Hadamard-type FDs in [66], and Hilfer FDs in [67].
- For the first time, DMMs were derived from equations with general FDs and FIs in [68,69].
- DMMs were obtained from equations with distributed-order FDs in [70].
- DMMs were obtained from equations with two FDs of arbitrary orders in [24,59,71].
- The first computer simulations of some such DMMs, which were obtained from nonlinear equations with FDs, are proposed in the papers [60,72,73].
- New types of chaotic behavior of systems with nonlocality in time were discovered in the papers [60,72], in the 2013 papers [74,75,76], and in the 2014 works [77,78].
- Note also new works on tunable subdiffusion in DMMs, by Mendez-Bermudez and Aguilar-Sanchez [79];scaling invariance analyses for DMMs, by Borin [80]; changes in the complexity of DMMs, by Orinaite, Telksniene, Telksnys, Ragulskis [81]; and Arnold tongues of divergence in Caputo DMMs, by Orinaite, Smidtaite, Ragulskis [82].

Note that in these works, the exact analytical solutions and DMMs from equations with FDs and FIs are derived without approximations. The importance of this approach to obtaining DMMs is that these maps are derived from the exact analytical solutions of nonlinear equations with FDs and FIs for a very wide class of nonlinearities without any approximations.

There are three methods for obtaining exact solutions of nonlinear equations with periodic kicks:

- A physical method that has been used in physics papers for ordinary differential equations of integer order and with kicks [4,5,6]. Attempts to generalize this method from equations with integer derivatives to fractional derivatives did not lead other scientists to positive results.
- A method based on the equivalence of the Cauchy-type problem for fractional differential equations and the Volterra integral equation of second kind, as reported in the 2009 papers [57,58]. In these papers, we use the results of the works [20,83,84,85,86,87]. A disadvantage of this second method which hinders generalization to other types of fractional derivatives and integrals is the fact that theorems with such equivalence have not been proven for all types of fractional derivatives.
- A method based on the use of the first and second fundamental theorems of fractional calculus, which is proposed in the 2021 papers [63,64,65,66,67]. Note that the term “fundamental theorems of fractional calculus” was not used until 2008. These important properties of operators were present in books as Lemmas (for example, see Lemmas 2.4, 2.5, 2.21, and 2.22 in [20]). The use of a term similar to classical calculus was proposed in the 2008 article [88] and became generally accepted with the 2020 work by Luchko [89] and the 2021 papers [35,36] about general fractional calculus. The fundamental theorems of FC allow us to obtain exact solutions and DMMs for different FDs and FIs, as generalized in [63,64,65,66,67] and subsequent articles by the author. Note that in the paper [71], some generalization of this method is proposed for equations with kicks and two FDs.

For physics and mechanics, the connection of discrete maps with equations with FDs and FIs is primarily important. Therefore, it is important to obtain DMMs from various equations with FDs and FIs without approximations. Recently some DMMs were proposed by using discrete FC [90,91,92] and discrete general FC [93,94,95]. Such maps, which are called “fractional difference maps”, were considered, for example, by Edelman in the 2015 works [96,97,98,99], in the 2018 papers [100,101], in the reviews [102,103], and in the paper by Edelman, Helman, and Smidtaite [104,105,106,107,108]. Unfortunately such fractional discrete maps are not associated with the exact solutions of equations with FDs, and such maps were not obtained from equations with FDs without approximations. In addition, there are no well-founded models in physics, mechanics, and economics that were described by the equation of discrete FC. Unfortunately, there are currently no studies on the relationship between discrete FC, described in [90,91,92], and classical FC [17,18,19,20,21,22,23]. However, the description of the new chaotic type of behavior of nonlinear systems with memory and new types of attractors of “fractional difference” maps is important. This gives hope that similar attractors and similar chaotic behavior are realized in discrete maps obtained from equations with FDs without approximations.

It is very important to generalize the proposed method and to derive the exact analytical solutions of nonlinear equations with FDs from the one-dimensional case to the multi-dimensional case. The first fractional generalization of the proposed method of obtaining exact analytical solutions and DMMs was proposed in the 2010 works [24,59]. In these works, the fractional generalization of the Henon and Zaslavsky maps, which are two-dimensional dissipative quadratic maps given by two coupled equations, is proposed. In the paper [60], the computer simulation of fractional Zaslavsky maps is realized. Recently, in the works [109,110,111,112], some multi-dimensional DMMs are proposed by using discrete FC [90,91,92]. Unfortunately these fractional discrete maps were proposed without any connection with equations with FDs or any differential equation at all. Therefore, these multi-dimensional DMMs cannot be considered the exact analytical solutions of nonlinear equations at discrete time points. Let us note that Orinaite, Smidtaite, and Ragulsk, in the 2025 paper [82], proposed to derive multi-dimensional DMMs as maps of matrices from the exact analytical solutions of nonlinear equations with FDs for with matrices. This OSR approach to multi-dimensional maps that are exact solutions of equations with FDs is very promising.

## 3. General Fractional Lorenz Systems with Memory

### 3.1. Generalization of Fractional Lorenz Systems

Let us consider the generalized Lorenz equations

(6) $$\left\{\begin{array}{cc} D_{\left(K_{x}\right)}^{t,*}x & =F_{L,x}\left(x,y,z\right),\\ D_{\left(K_{y}\right)}^{t,*}y & =F_{L,y}\left(x,y,z\right),\\ D_{\left(K_{z}\right)}^{t,*}z & =F_{L,z}\left(x,y,z\right), \end{array}\right.$$

where

(7) $$D_{\left(K_{j}\right)}^{t,*}x_{j}\;:=\;\int_{0}^{t}K_{j}\left(t-s\right)\;x_{j}^{\left(1\right)}\left(s\right)\;ds$$

with $x_{1}=x$, $x_{2}=y$, $x_{3}=z$, and

(8) $$\left\{\begin{array}{cc} F_{L,x}\left(x,y,z\right) & =\sigma (y-x),\\ F_{L,y}\left(x,y,z\right) & =x(\rho -z)-y,\\ F_{L,z}\left(x,y,z\right) & =xy-\beta z. \end{array}\right.$$

If we use the functions

(9) $$\left\{\begin{array}{cc} F_{R,x}\left(x,y,z\right) & =-y-z\\ F_{R,y}\left(x,y,z\right) & =x+ay\\ F_{R,z}\left(x,y,z\right) & =b+z(x-c) \end{array}\right.$$

in Equation (6) instead of functions (8), then we get the well-known Rössler system [9,10].

If we use the functions

(10) $$\left\{\begin{array}{cc} F_{C,x}\left(x,y,z\right) & =a(y-z)\\ F_{C,y}\left(x,y,z\right) & =(c-a)x+cy-xz\\ F_{C,z}\left(x,y,z\right) & =-bz+xy \end{array}\right.$$

in Equation (6) instead of functions (8), then we get the Chen system [13,14].

It is also possible to consider generalizations of other Lorenz-type systems that are described in Petras’ works [15,16].

Let us consider some special cases of Lorenz-type system (6) with (8).

(1) If all kernels $K_{j}\left(t-s\right)$ are described by the Dirac delta-function $K_{j}\left(t-s\right)=\delta \left(t-s\right)$, then Equation (6) gives the standard equations of the Lorenz system [7] in the form

(11) $$\left\{\begin{array}{cc} \frac{dx}{dt} & =\sigma (y-x),\\ \frac{dy}{dt} & =x(\rho -z)-y,\\ \frac{dz}{dt} & =xy-\beta z. \end{array}\right.$$

Lorenz Equation (11) are ordinary differential equations of the first order.

(2) If all kernels $K_{j}\left(t-s\right)$ have the form

(12) $$K_{j}\left(t-s\right)\;=\;h_{n-\alpha_{j}}\left(t\right)$$

where $\alpha_{j}\in \left(n-1,n\right]$ with n∈N and

(13) $$h_{\alpha }\left(t\right)\;:=\;\frac{t^{\alpha -1}}{\Gamma (\alpha )}\;\left(\alpha \;>\;0\right),$$

where Γ(z) is the gamma function [20], then Equation (6) gives the fractional Lorenz system [12,15,16] that is described by equations with Caputo fractional derivatives of order $\alpha_{j}$ [20].

(3) It is possible not to limit oneself only to the power function in (13) and to consider a wide class of functions $K_{j}\left(t\right)$. From a physical point of view, functions $K_{j}\left(t\right)$ are memory functions. Therefore, in applications, it is important to consider a wider class of memory functions. From a mathematical point of view, functions $K_{j}\left(t\right)$ are operator kernels. In this case, for the self-consistency of the mathematical calculus of such operators, it is very important that operator (7) has the integral operator

(14) $$I_{\left(M_{j}\right)}^{t}x_{j}\;:=\;\int_{0}^{t}M_{j}\left(t-s\right)\;x_{j}\left(s\right)\;ds,$$

and that some analogs of the first and second fundamental theorems of fractional calculus are satisfied. For the existence of generalized fundamental theorems, operator kernels $K_{j}\left(t\right)$ and $M_{j}\left(t\right)$ must satisfy certain conditions. For example, kernels $K_{j}\left(t\right)$ and $M_{j}\left(t\right)$ must satisfy the Sonin condition,

(15) $$\int_{0}^{t}M\left(t-s\right)\;K\left(s\right)\;ds\;=\;1$$

for all t∈(0,∞), and the Luchko condition,

(16) $$M\left(t\right),K\left(t\right)\in C_{-1,0}\left(0,\infty \right),$$

where

(17) $$C_{a,b}\left(0,\infty \right)\;=\;\left\{g\left(t\right):\;g\left(t\right)\notin C\left[0,\infty \right),\;g\left(t\right)=t^{p}\;h\left(t\right),\;h\left(t\right)\in C\left[0,\infty \right),\;t>0,\;a<p<b\right\}.$$

The set of such pairs $\left(K_{j}\left(t\right),M_{j}\left(t\right)\right)$ of memory functions that satisfy conditions (15) and (16) is denoted by $L_{1}$. Note that it is these conditions (15) and (16) that ensure the existence of fundamental theorems for operators (7) and (14). Therefore, operators (14) and (7) are called the general fractional derivative (GFD) and the general fractional integral (GFI).

The GFI is defined by the equation

(18) $$\left(I_{\left(M\right)}^{t}X\right)\left(t\right)\;=\;I_{\left(M\right)}^{t}\left[s\right]X\left(s\right)\;=\;\int_{0}^{t}ds\;M\left(t-s\right)\;X\left(s\right)$$

for t∈(0,∞), where it is assumed that $X\left(t\right)\in C_{-1}\left(0,\infty \right)=C_{-1,\infty }\left(0,\infty \right)$ and $(M\left(t\right),K\left(t\right)(\in L_{1}$.

The GFD of the Caputo type is defined as

(19) $$\left(D_{\left(K\right)}^{t,*}X\right)\left(t\right)\;=\;D_{\left(K\right)}^{t,*}\left[s\right]X\left(s\right)\;=\;I_{\left(K\right)}^{t}\left[s\right]X^{\left(1\right)}\left(s\right)\;=\;\int_{0}^{t}ds\;K\left(t-s\right)\;X^{\left(1\right)}\left(s\right),$$

and the GFD of the Riemann–Liouville type is defined as

(20) $$\left(D_{\left(K\right)}^{t}X\right)\left(t\right)\;=\;D_{\left(K\right)}^{t}\left[s\right]X\left(s\right)\;=\;\frac{d}{dt}I_{\left(K\right)}^{t}\left[s\right]X\left(s\right)\;=\;\frac{d}{dt}\int_{0}^{t}ds\;K\left(t-s\right)\;X\left(s\right)$$

for t∈(0,∞), where it is assumed that $X^{\left(1\right)}\left(t\right)\in C_{-1}\left(0,\infty \right)=C_{-1,\infty }\left(0,\infty \right)$ and $\left(M\left(t\right),K\left(t\right)\right)\in L_{1}$. Note that FD (20) can be expressed through FD (19) as

(21) $$\left(D_{\left(K\right)}^{t}X\right)\left(t\right)\;=\;\left(D_{\left(K\right)}^{t,*}X\right)\left(t\right)\;+\;X\left(0\right)\;K\left(t\right).$$

Therefore, we will derive in detail the solution of the equations and DMMs only for GFDs of the Caputo type.

The calculus of operators (7) and (14) that are used for these conditions is called general fractional calculus (GFC) of the Luchko form. GFC of the Luchko form was proposed by Yuri Luchko in the 2021 papers [35,36,37]. Note that GFC of the Luchko form is the most convenient form of GFC for applications. These GFIs and GFDs satisfy the fundamental theorems of GFC that are proved in [35,36].

### 3.2. Matrix Form of GF Lorenz-Type Equations

To simplify the equations when deriving solutions to a nonlinear equation of a generalized Lorenz-type system with kicks, we introduce some matrix-type notations.

Let us define the matrices

(22) $$X\left(t\right)\;:=\;\left(\begin{array}{c} x(t)\\ y(t)\\ z(t) \end{array}\right),\;F_{L}\left(x,y,z\right)\;:=\;\left(\begin{array}{c} F_{L,x}\left(x,y,z\right)\\ F_{L,y}\left(x,y,z\right)\\ F_{L,z}\left(x,y,z\right) \end{array}\right)\;I=\left(\begin{array}{ccc} 1 & 0 & 0\\ 0 & 1 & 0\\ 0 & 0 & 1 \end{array}\right),$$

and the matrix of memory functions

$$M\left(t\right)=\left(\begin{array}{ccc} M_{x}\left(t\right) & 0 & 0\\ 0 & M_{y}\left(t\right) & 0\\ 0 & 0 & M_{z}\left(t\right) \end{array}\right)\;K\left(t\right)=\left(\begin{array}{ccc} K_{x}\left(t\right) & 0 & 0\\ 0 & K_{y}\left(t\right) & 0\\ 0 & 0 & K_{z}\left(t\right) \end{array}\right).$$

The product of matrices M(t−s) and K(s) is standard,

$$M\left(t-s\right)\;K\left(s\right)\;=\;\left(\begin{array}{ccc} M_{x}\left(t-s\right) & 0 & 0\\ 0 & M_{y}\left(t-s\right) & 0\\ 0 & 0 & M_{z}\left(t-s\right) \end{array}\right)\;\left(\begin{array}{ccc} K_{x}\left(s\right) & 0 & 0\\ 0 & K_{y}\left(s\right) & 0\\ 0 & 0 & K_{z}\left(s\right) \end{array}\right)\;=\;$$

(23) $$\left(\begin{array}{ccc} M_{x}\left(t-s\right)\;K_{x}\left(s\right) & 0 & 0\\ 0 & M_{y}\left(t-s\right)\;K_{y}\left(s\right) & 0\\ 0 & 0 & M_{z}\left(t-s\right)\;K_{z}\left(s\right) \end{array}\right),$$

and the product of matrices M(t−s) and X(s) is

$$M\left(t-s\right)\;X\left(s\right)\;=\;\left(\begin{array}{ccc} M_{x}\left(t-s\right) & 0 & 0\\ 0 & M_{y}\left(t-s\right) & 0\\ 0 & 0 & M_{z}\left(t-s\right) \end{array}\right)\;\left(\begin{array}{c} x(t)\\ y(t)\\ z(t) \end{array}\right)\;=\;$$

(24) $$\left(\begin{array}{ccc} M_{x}\left(t-s\right)\;x\left(t\right) & 0 & 0\\ 0 & M_{y}\left(t-s\right)\;y\left(t\right) & 0\\ 0 & 0 & M_{z}\left(t-s\right)\;z\left(t\right) \end{array}\right).$$

The product of matrices K(t−s) and X(s) is written similarly.

The integration of the resulting matrix M(t−s)K(s) has the form

$$\int_{0}^{t}M\left(t-s\right)\;K\left(s\right)\;ds\;=\;$$

(25) $$\left(\begin{array}{ccc} \int_{0}^{t}M_{x}\left(t-s\right)\;K_{x}\left(s\right)\;ds & 0 & 0\\ 0 & \int^{t}M_{y}\left(t-s\right)\;K_{y}\left(s\right)\;ds & 0\\ 0 & 0 & \int_{0}^{t}M_{z}\left(t-s\right)\;K_{z}\left(s\right)\;ds \end{array}\right).$$

Therefore, the Sonin condition for the matrices of memory functions M(t) and K(s) has the form

(26) $$\int_{0}^{t}M\left(t-s\right)\;K\left(s\right)\;ds\;=\;H\left(t\right)\;:=\;\theta \left(t\right)\;I,$$

where θ(t) is the Heaviside step function and *I* is the identity matrix.

The GFI in matrix form is defined as

$$I_{\left(M\right)}^{t}\left[s\right]X\left(s\right)\;=\;\int_{0}^{t}ds\;M\left(t-s\right)\;X\left(s\right)\;=\;\int_{0}^{t}ds\;\left(\begin{array}{c} M_{x}\left(t-s\right)\;x\left(s\right)\\ M_{y}\left(t-s\right)\;y\left(s\right)\\ M_{z}\left(t-s\right)\;z\left(s\right) \end{array}\right)\;=\;$$

(27) $$\left(\begin{array}{c} \int_{0}^{t}ds\;M_{x}\left(t-s\right)\;x\left(s\right)\\ \int_{0}^{t}ds\;M_{y}\left(t-s\right)\;y\left(s\right)\\ \int_{0}^{t}ds\;M_{z}\left(t-s\right)\;z\left(s\right) \end{array}\right)\;=\;\left(\begin{array}{c} I_{\left(M_{x}\right)}^{t}\left[s\right]x\left(s\right)\\ I_{\left(M_{y}\right)}^{t}\left[s\right]y\left(s\right)\\ I_{\left(M_{z}\right)}^{t}\left[s\right]z\left(s\right) \end{array}\right).$$

The GFD of the Caputo type in matrix form is

$$D_{\left(K\right)}^{t,*}\left[s\right]X\left(s\right)\;=\;\int_{0}^{t}ds\;K\left(t-s\right)\;X^{\left(1\right)}\left(s\right)\;=\;\int_{0}^{t}ds\;\left(\begin{array}{c} K_{x}\left(t-s\right)\;x^{\left(1\right)}\left(s\right)\\ K_{y}\left(t-s\right)\;y^{\left(1\right)}\left(s\right)\\ K_{z}\left(t-s\right)\;z^{\left(1\right)}\left(s\right) \end{array}\right)\;=\;$$

(28) $$\left(\begin{array}{c} \int_{0}^{t}ds\;K_{x}\left(t-s\right)\;x^{\left(1\right)}\left(s\right)\\ \int_{0}^{t}ds\;K_{y}\left(t-s\right)\;y^{\left(1\right)}\left(s\right)\\ \int_{0}^{t}ds\;K_{z}\left(t-s\right)\;z^{\left(1\right)}\left(s\right) \end{array}\right)\;=\;\left(\begin{array}{c} D_{\left(K_{x}\right)}^{t,*}\left[s\right]x\left(s\right)\\ D_{\left(K_{y}\right)}^{t,*}\left[s\right]y\left(s\right)\\ D_{\left(K_{z}\right)}^{t,*}\left[s\right]z\left(s\right) \end{array}\right).$$

Note that memory functions $K_{x}\left(t\right)$, $K_{y}\left(t\right)$, and $K_{z}\left(t\right)$ may be of different types in the general case.

As a result, the general fractional (GF) Lorenz-type system that is described by the system of equations in (6) can be rewritten in the matrix form

(29) $$D_{\left(K\right)}^{t,*}X\left(t\right)\;=\;F_{L}\left(x\left(t\right),y\left(t\right),z\left(t\right)\right),$$

where

(30) $$D_{\left(K\right)}^{t,*}X\left(t\right)\;:=\;\int_{0}^{t}K\left(t-s\right)\;X^{\left(1\right)}\left(s\right)\;ds.$$

For such GF Lorenz-type systems, it is interesting to study how the chaotic behavior and strange attractors change when using non-power-law memory functions compared with the results for power-law memory [15,16]. This topic is not the subject of this paper. In the next section, Equation (29) is changed to describe the kicked form of GF Lorenz-type systems.

It should be emphasized that the use of matrix notation allows us to consider not only any GF generalization of Lorenz-type systems [15,16] but also multi-dimensional systems of any dimension that are described by nonlinear equations with GFDs and with different sets of memory functions.

## 4. Kicked GF Lorenz-Type Systems with Memory

### 4.1. Equations of Kicked GF Lorenz-Type Systems

Let us consider a nonlinear equation with a GFD and periodic kicks

(31) $$D_{\left(K\right)}^{t,*}X\left(t\right)\;=\;A\;F_{L}\left(x\left(t\right),y\left(t\right),z\left(t\right)\right)\sum_{k=1}^{\infty }\delta \left(t\right)/\tau -k),$$

where $D_{\left(K\right)}^{t,*}$ is GFD (19), τ>0 is the period of the kicks, A is the amplitude of the kicks, δ(t) is the Dirac delta-function, and $F_{L}\left(x_{1},x_{2},x_{3}\right)$ is the real-valued continuous function that describes the Lorenz-type system.

Equation (31) contains the delta-functions, which are generalized functions [20,113,114]. Therefore, the product

(32) $$F_{L}\left(x\left(t\right),y\left(t\right),z\left(t\right)\right)\;\delta \left(t/\tau -k\right)$$

exists if the function $F_{L}^{*}\left(t\right):=F_{L}\left(x\left(t\right),y\left(t\right),z\left(t\right)\right)$ is a continuous function at the points $t_{k}\;=\;k\;\tau$ with integer k>0. [20,113,114]. However, the solution X(t) of Equation (31) is discontinuous: $X\left(t_{k}-0\right)\neq X\left(t_{k}+0\right)$; therefore, the function $F_{L}\left(t\right)$ is a discontinuous function at the points $t_{k}\;=\;k\;\tau$, i.e., $F_{L}\left(X\left(t_{k}-0\right)\right)\neq F_{L}\left(X\left(t_{k}+0\right)\right)$. This means that product (32) in Equation (31) is defined incorrectly.

To correctly define product (32) in Equation (31), we use t−η with η>0 in the argument of the function $F_{L}\left(x_{1}\left(t\right),x_{2}\left(t\right),x_{3}\left(t\right)\right)$ [96]. We take into account that the discrete map is defined for the function

(33) $$X_{k}\;=\;X\left(kT-0\right)\;=\;\lim_{\eta \to 0+}X\left(kT-\eta \right)$$

to make a sense of product (32) and have the correct definition (33).

The function $F_{L}^{*}\left(t-\eta \right)$ will be continuous in the neighborhood $\left(t_{k}-\epsilon ,t_{k}+\epsilon \right)$ of the point $t_{k}=k\tau$ if η>ϵ, that is, if $t_{k}-\eta \notin \left(t_{k}-\epsilon ,t_{k}+\epsilon \right)$ and if $F_{L}\left(x,y,z\right)$ is continuous in its domain $\Omega \subseteq R^{n}$ of definition. Therefore, we assume that

(34) 0<ϵ<η≪τ.

In this case, Equation (31) with $F_{L}\left(X\left(t-\eta \right)\right)$ instead of $F_{L}\left(X\left(t\right)\right)$ takes the mathematically correct form.

As a result, a kicked GF Lorenz-type system (KGF-LTS) is described by the equation

(35) $$D_{\left(K\right)}^{t,*}X\left(t\right)\;=\;A\;F_{L,\eta }\left(x\left(t\right),y\left(t\right),z\left(t\right)\right)\sum_{k=1}^{\infty }\delta \left(t/\tau -k\right),$$

where

(36) $$F_{L,\eta }\left(x\left(t\right),y\left(t\right),z\left(t\right)\right)\;:=\;F_{L}\left(x\left(t-\eta \right),y\left(t-\eta \right),z\left(t-\eta \right)\right),$$

and 0<ϵ<η≪τ.

### 4.2. Derivation of Exact Analytical Solutions

The derivation of exact analytical solutions of nonlinear equations with GFDs is based on the second fundamental theorem of GFC and the properties of GFDs and GFIs. By using the exact solution at discrete moments of time and the difference between these solutions at neighboring time points, we derive GF Lorenz-type discrete maps with memory.

Let us derive the solution of the equation

(37) $$D_{\left(K\right)}^{t,*}\left[s\right]\;X\left(s\right)\;=\;A\;F_{L,\eta }\left(x\left(t\right),y\left(t\right),z\left(t\right)\right)\sum_{k=1}^{\infty }\delta \left(t/\tau -k\right),$$

where we assume that $\left(K\left(t\right),M\left(t\right)\right)\in L_{1}$ and $X\left(t\right)\in C_{-1}^{1}\left(0,\infty \right)$.

By applying GFI (18) to Equation (37), we get

(38) $$I_{\left(M\right)}^{u}\left[t\right]\;\left(D_{\left(K\right)}^{t,*}\left[s\right]\;X\left(s\right)\right)\;=\;A\;I_{\left(M\right)}^{u}\left[t\right]\;\left(F_{L,\eta }\left(x\left(t\right),y\left(t\right),z\left(t\right)\right)\sum_{k=1}^{\infty }\delta \left(\left(t-\epsilon \right)/\tau -k\right)\right),$$

where u>t>s>0. Using the second fundamental theorem of GFC in the form

(39) $$I_{\left(M\right)}^{u}\left[t\right]D_{\left(K\right)}^{t,*}\left[s\right]\;X\left(s\right)\;=\;X\left(u\right)\;-\;X\left(0\right)$$

which holds for $X\left(t\right)\in C_{-1}^{1}\left(0,\infty \right)$ (see Theorem 4 in [35] and Theorem 2 in [36]), we derive

(40) $$X\left(u\right)\;-\;X\left(0\right)\;=\;A\;I_{\left(M\right)}^{u}\left[t\right]\;\left(F_{L,\eta }\left(x\left(t\right),y\left(t\right),z\left(t\right)\right)\sum_{k=1}^{\infty }\delta \left(t/\tau -k\right)\right).$$

Using (18), Equation (40) is written as

(41) $$X\left(u\right)\;-\;X\left(0\right)\;=\;A\;\int_{0}^{u}dt\;M\left(u-t\right)\;F_{L,\eta }\left(x\left(t\right),y\left(t\right),z\left(t\right)\right)\sum_{k=1}^{\infty }\delta \left(t/\tau -k\right).$$

For τn<u<τ(n+1), Equation (41) gives

(42) $$X\left(u\right)\;-\;X\left(0\right)\;=\;A\;\sum_{k=1}^{n}\int_{0}^{u}dt\;M\left(u-t\right)\;F_{L,\eta }\left(x\left(t\right),y\left(t\right),z\left(t\right)\right)\delta \left(t/\tau -k\right).$$

Using the equality

(43) $$\int_{0}^{u}dt\;f\left(t\right)\delta \left(t/\tau -k\right)\;=\;\tau \;f\left(k\right),$$

which holds for and 0<k<u, with f(t) being a continuous function in the neighborhood of point $t_{k}=k\tau$, Equation (42) gives

(44) $$X\left(u\right)\;-\;X\left(0\right)\;=\;A\;\tau \;\sum_{k=1}^{n}M\left(u-k\tau \right)\;F_{L,\eta }\left(x\left(\tau k\right),y\left(\tau k\right),z\left(\tau k\right)\right).$$

As a result, for τn<t<τ(n+1), Equation (37) has the solution

(45) $$X\left(t\right)\;=\;X\left(0\right)\;+\;A\;\tau \;\sum_{k=1}^{n}M\left(t-\tau k\right)\;F_{L,\eta }\left(x\left(\tau k\right),y\left(\tau k\right),z\left(\tau k\right)\right).$$

For t∈(0,τ(n+1)), Equation (37) has the solution

(46) $$X\left(t\right)\;=\;X\left(0\right)\;+\;A\;\tau \;\sum_{k=1}^{n}M\left(t-\tau k\right))\;F_{L,\eta }\left(x\left(\tau k\right),y\left(\tau k\right),z\left(\tau k\right)\right)\;H\left(t-k\tau \right),$$

where H(t−kτ) is the matrix Heaviside step function and

(47) $$F_{L,\eta }\left(x\left(\tau k\right),y\left(\tau k\right),z\left(\tau k\right)\right)\;:=\;F_{L}\left(x\left(\tau k-\eta \right),y\left(\tau k-\eta \right),z\left(\tau k-\eta \right)\right).$$

The use of Riemann–Liouville FD (20) instead of Caputo FD (19) in Equation (35) gives

(48) $$D_{\left(K\right)}^{t}\left[s\right]\;X\left(s\right)\;=\;A\;F_{L,\eta }\left(x\left(t\right),y\left(t\right),z\left(t\right)\right)\sum_{k=1}^{\infty }\delta \left(t/\tau -k\right).$$

For τn<t<τ(n+1), Equation (48) has the exact analytical solution

(49) $$X\left(t\right)\;=\;A\;\tau \;\sum_{k=1}^{n}M\left(t-\tau k\right)\;F_{L,\eta }\left(x\left(\tau k\right),y\left(\tau k\right),z\left(\tau k\right)\right).$$

Note that Equation (49) differs from Equation (45) by the absence of the term X(0).

### 4.3. Derivation of Lorenz-Type Discrete Maps from Exact Solution

Discrete maps are derived from exact analytical solution (45) of Equation (37) for the discrete time points t=τn+ϵ and t=τ(n+1)+ϵ with integer n>0.

Let us define the function $X_{k}$ as X(t) for the time points t=τk+η at η→0+ such that

(50) $$X_{k}\;:=\;\lim_{\eta \to 0+}X\left(\tau k-\eta \right),\;\left(k=1,\ldots ,n+1\right)$$

with η>0.

Solution (45) of Equation (37) for t=τ(n+1)−η, which belongs to the interval (τn,τ(n+1)), since 0<η≪τ, is written as

(51) $$X\left(\tau \left(n+1\right)-\eta \right)\;=\;X\left(0\right)\;+\;A\;\sum_{k=1}^{n}M\left(\tau \left(n+1\right)-\tau k-\eta \right)\;F_{L,\eta }\left(x\left(\tau k\right),y\left(\tau k\right),z\left(\tau k\right)\right),$$

(52) $$X\left(\tau n-\eta \right)\;=\;X\left(0\right)\;+\;A\;\sum_{k=1}^{n-1}M\left(\tau n-\tau k-\eta \right)\;F_{L,\eta }\left(x\left(\tau k\right),y\left(\tau k\right),z\left(\tau k\right)\right).$$

Using that $M\left(t\right)\in C_{-1}\left(0,\infty \right)$, we get

(53) $$\lim_{\eta \to 0+}M\left(t-\eta \right)\;=\;M\left(t\right)$$

for t>0.

By using property (53) and functions (50), solutions (51) and (52) at the limit η→0+ give

(54) $$X_{n+1}=X\left(0\right)\;+\;A\;\tau \;\sum_{k=1}^{n}M\left(\tau \left(n-k+1\right)\right)\;F_{L}\left(x_{k},y_{k},z_{k}\right),$$

(55) $$X_{n}=X\left(0\right)\;+\;A\;\tau \;\sum_{k=1}^{n-1}M\left(\tau \left(n-k\right)\right)\;F_{L}\left(x_{k},y_{k},z_{k}\right),$$

where we use

$$\lim_{\eta \to 0+}F_{L,\eta }\left(x\left(\tau k\right),y\left(\tau k\right),z\left(\tau k\right)\right)\;=\;$$

(56) $$\lim_{\eta \to 0+}F_{L}(x\left(\tau k-\eta \right),y\left(\tau k-\eta \right),z\left(\tau k-\eta \right)\;=\;F_{L}\left(x_{k},y_{k},z_{k}\right).$$

The subtraction of Equation (55) from Equation (54) gives

$$X_{n+1}\;-\;X_{n}\;=\;A\;\tau \;M\left(\tau \right)\;F_{L}\left(x_{n},y_{n},z_{n}\right)\;+\;$$

(57) $$A\;\tau \;\sum_{k=1}^{n-1}\left(M(\tau (n-k+1))-M(\tau (n-k))\right)\;F_{L}\left(x_{k},y_{k},z_{k}\right).$$

As a result, the GF Lorenz-type discrete map with memory is

(58) $$X_{n+1}\;=\;X_{n}\;+\;A\;\tau \;M\left(\tau \right)\;F_{L}\left(x_{n},y_{n},z_{n}\right)\;+\;A\;\tau \;\sum_{k=1}^{n-1}L\left(\tau ,n-k\right)\;F_{L}\left(x_{k},y_{k},z_{k}\right),$$

where

(59) L(τ,m):=M(τ(m+1))−M(τm),

where (M(t),K(t)) is the pair of memory functions from the set $L_{1}$.

Note that the map that is derived from exact solution (49) of Equation (48) with the Riemann–Liouville GFD is

(60) $$X_{n+1}\;=\;X_{n}+A\;\tau \;M\left(\tau \right)\;F_{L}\left(x_{n},y_{n},z_{n}\right)\;+\;A\;\tau \;\sum_{k=1}^{n-1}L\left(\tau ,n-k\right)\;F_{L}\left(x_{k},y_{k},z_{k}\right),$$

which coincides with map (58).

### 4.4. Examples of First Steps of Lorenz-Type Discrete Maps

Equation (58) is the exact analytical solution at the discrete time points t=τk with integer k>0. Let us give the examples of Equation (58) and the examples of the functions L(τ,n) with n=1, n=2, and n=3 for Lorenz form (8) of $F_{L}\left(x,y,z\right)$.

(1) For n=1, Equation (58), is

(61) $$X_{2}\;=\;X_{1}\;+\;A\;\tau \;M\left(\tau \right)\;F_{L}\left(x_{1},y_{1},z_{1}\right).$$

Using the notations *x*, *y*, and *z*, matrix Equation (61) gives

(62) $$\left\{\begin{array}{cc} x_{2}\;=\; & x_{1}\;+\;A\;\tau \;M\left(\tau \right)\;\sigma \left(y_{1}-x_{1}\right),\\ y_{2}\;=\; & y_{1}\;+\;A\;\tau \;M\left(\tau \right)\;\left(x_{1}\left(\rho -z_{1}\right)-y_{1}\right),\\ z_{2}\;=\; & z_{1}\;+\;A\;\tau \;M\left(\tau \right)\;\left(x_{1}y_{1}-\beta z_{1}\right), \end{array}\right.$$

where τ>0 is the period of the kicks and A is the amplitude of the kicks.

(2) For n=2, Equation (58) is

(63) $$X_{3}\;=\;X_{2}\;+\;A\;\tau \;M\left(\tau \right)\;F_{L}\left(x_{2},y_{2},z_{2}\right)\;+\;A\;\tau \;L\left(\tau ,1\right)\;F_{L}\left(x_{1},y_{1},z_{1}\right).$$

where

(64) L(τ,1)=M(2τ)−M(τ).

Using the notations *x*, *y*, and *z*, matrix Equation (63) gives

(65) $$\left\{\begin{array}{cc} x_{3}\;=\; & x_{2}\;+\;A\;\tau \;M\left(\tau \right)\;\sigma \left(y_{2}-x_{2}\right)\;+\;A\;\tau \;L\left(\tau ,1\right)\;\left(\sigma \left(y_{1}-x_{1}\right)\right),\\ y_{3}\;=\; & y_{2}\;+\;A\;\tau \;L\left(\tau ,1\right)\;\left(x_{2}\left(\rho -z_{2}\right)-y_{2}\right)\;+\;A\;\tau \;L\left(\tau ,1\right)\;\left(x_{1}\left(\rho -z_{1}\right)-y_{1}\right),\\ z_{3}\;=\; & z_{2}\;+\;A\;\tau \;M\left(\tau \right)\;\left(x_{2}y_{2}-\beta z_{2}\right)\;+\;A\;\tau \;L\left(\tau ,1\right)\;\left(x_{1}y_{1}-\beta z_{1}\right), \end{array}\right.$$

where L(τ,1) is defined by Equation (64).

(3) For n=3, Equation (58) is

$$X_{4}\;=\;X_{3}\;+\;A\;\tau \;M\left(\tau \right)\;F_{L}\left(x_{3},y_{3},z_{3}\right)\;+\;$$

(66) $$A\;\tau \;L\left(\tau ,2\right)\;F_{L}\left(x_{1},y_{1},z_{1}\right)\;+\;A\;\tau \;L\left(\tau ,1\right)\;F_{L}\left(x_{2},y_{2},z_{2}\right).$$

where

(67) L(τ,1)=M(2τ)−M(τ),L(τ,2)=M(3τ)−M(2τ).

Using the notations *x*, *y*, and *z*, matrix Equation (66) gives

$$x_{4}\;=\;x_{3}\;+\;A\;\tau \;M\left(\tau \right)\;\sigma \left(y_{3}-x_{3}\right)\;+\;$$

(68) $$A\;\tau \;L\left(\tau ,2\right)\;\left(\sigma \left(y_{1}-x_{1}\right)\right)\;+\;A\;\tau \;L\left(\tau ,1\right)\;\left(\sigma \left(y_{2}-x_{2}\right)\right).$$

$$y_{4}\;=\;y_{3}\;+\;A\;\tau \;M\left(\tau \right)\;\left(x_{3}\left(\rho -z_{3}\right)-y_{3}\right)\;+\;$$

(69) $$A\;\tau \;L\left(\tau ,2\right)\;\left(x_{1}\left(\rho -z_{1}\right)-y_{1}\right)\;+\;A\;\tau \;L\left(\tau ,1\right)\;\left(x_{2}\left(\rho -z_{2}\right)-y_{2}\right).$$

$$z_{4}\;=\;z_{3}\;+\;A\;\tau \;M\left(\tau \right)\;\left(x_{3}y_{3}-\beta z_{3}\right)\;+\;$$

(70) $$A\;\tau \;L\left(\tau ,2\right)\;\left(x_{1}y_{1}-\beta z_{1}\right)\;+\;A\;\tau \;L\left(\tau ,1\right)\;\left(x_{2}y_{2}-\beta z_{2}\right).$$

### 4.5. Examples of Memory Functions

Let us give examples of various memory functions $\left(M_{j}\left(t\right),\;K_{j}\left(t\right)\right)$ that belong to the set $L_{1}$ and can be used in GF Lorenz-type discrete maps:

- The memory function pair of the power-law type is
  (71) $$M_{PL}\left(t\right)\;=\;h_{\alpha }\left(t\right)\;:=\;\frac{t^{\alpha -1}}{\Gamma (\alpha )},\;K_{PL}\left(t\right)\;=\;h_{1-\alpha }\left(t\right),$$
  where α∈(0,1) and
  (72) $$L_{PL}\left(\tau ,m\right)\;=\;h_{\alpha }\left(\tau \right)\left({\left(m+1\right)}^{\alpha -1}\;-\;m^{\alpha -1}\right).$$
- The memory function pair of the gamma-distribution type is
  (73) $$M_{GD}\left(t\right)\;=\;h_{\alpha ,\rho }\left(t\right)\;:=\;\frac{t^{\alpha -1}}{\Gamma (\alpha )}e^{-\;\rho \;t},\;K_{GD}\left(t\right)\;=\;h_{1-\alpha ,\rho }\left(t\right)\;+\;\gamma_{1-\alpha }\left(t\right),$$
  where $\gamma_{\alpha }\left(t\right)\;:=\;\gamma \left(\alpha ,t\right)/\Gamma \left(\alpha \right)$ and γ(α,t) is the incomplete gamma function [115], α∈(0,1), β>0, and
  (74) $$L_{GD}\left(\tau ,m\right)\;=\;h_{\alpha ,m\rho }\left(\tau \right)\left({\left(m+1\right)}^{\alpha -1}\;e^{-\rho \tau }\;-\;m^{\alpha -1}\right).$$
- The memory function pair of the Mittag–Leffler type is
  (75) $$M_{ML}\left(t\right)\;=\;e_{\alpha ,\beta ,-1}\left(t\right)\;:=\;t^{\beta -1}\;E_{\alpha ,\beta }\left[-\;t^{\alpha }\right],\;K_{ML}\left(t\right)\;=\;h_{1+\alpha -\beta }\left(t\right)\;+\;h_{1-\beta }\left(t\right),$$
  where $E_{\alpha ,\beta }\left[z\right]$ is the two-parameter Mittag–Leffler function [20,116], 0<α≤β<1, and
  (76) $$L_{ML}\left(\tau ,m\right)\;=\;e_{\alpha ,\beta ,-1}\left(\tau \left(m+1\right)\right)\;-\;e_{\alpha ,\beta ,-1}\left(\tau m\right).$$
- The memory function pair of the Bessel function type is
  (77) $$M_{BF}\left(t\right)\;=\;\omega_{\alpha }\left(t\right)\;:=\;{\left(\sqrt{t}\right)}^{\alpha -1}\;J_{\alpha -1}\left(2\sqrt{t}\right),\;K_{BF}\left(t\right)\;=\;i^{\alpha -1}\;\omega_{\alpha }\left(i\;x\right),$$
  where the function $J_{\alpha }\left(t\right)$ is the Bessel function [20] and
  (78) $$L_{BF}\left(\tau ,m\right)\;=\;\omega_{\alpha }\left(\tau \left(m+1\right)\right)\;-\;\omega_{\alpha }\left(\tau m\right).$$
- The memory function pair of the hypergeometric type is
  (79) $$M_{HG}\left(t\right)\;=\;\phi_{\alpha ,\beta }\left(t\right)\;:=\;t^{\beta -1}\Phi \left(\alpha ,\beta ;-\;t\right)\;K_{HG}\left(t\right)\;=\;\frac{\sin(\pi \beta )}{\pi }\;\phi_{\alpha ,1-\beta }\left(t\right),$$
  where Φ(α;β;t)=F11(α;β;t) is the confluent hypergeometric Kummer function [20] and
  (80) $$L_{BF}\left(\tau ,m\right)\;=\;\phi_{\alpha ,\beta }\left(\tau \left(m+1\right)\right)\;-\;\phi_{\alpha ,\beta }\left(\tau m\right).$$

Let us give some remarks.

(1) For each pair (M(t),K(t)) of examples (71)–(79), one can use the pairs of the memory function $\left(M_{new}\left(t\right)=K\left(t\right),K_{new}\left(t\right)=M\left(t\right)\right)$. For example, in addition to memory function pair (75), we can also use the functions

(81) $$M_{ML,new}\left(t\right)\;=\;h_{1+\alpha -\beta }\left(t\right)\;+\;h_{1-\beta }\left(t\right),\;MK_{ML,new}\left(t\right)\;=\;e_{\alpha ,\beta ,-1}\left(t\right),$$

and

(82) $$L_{ML,new}\left(\tau ,m\right)\;=\;h_{1+\alpha -\beta }\left(\tau \right)\left({\left(m+1\right)}^{\alpha -\beta }\;-\;m^{\alpha -\beta }\right)\;+\;h_{1-\beta }\left(\tau \right)\left({\left(m+1\right)}^{-\beta }\;-\;m^{-\beta }\right),$$

where 0<α≤β<1.

(2) Note that all parameters in memory functions (71)–(79) are dimensionless quantities. To have standard physical dimensions in applications of GFC, we must use the pairs (M(λt),λK(λt)), with the parameter λ≠0, where the physical dimension is inversely related to time $\left[\lambda \right]=\left[t^{-1}\right]$.

(3) In a kicked GF Lorenz-type system, the functions $M_{x}\left(t\right)$, $M_{y}\left(t\right)$, and $M_{z}\left(t\right)$ can be of different types and/or with different parameter values. For example, one can use different types of memory functions, such as

(83) $$M_{x}\left(t\right)\;=\;h_{\alpha_{1}}\left(t\right),\;M_{x}\left(t\right)\;=\;e_{\alpha_{2},\beta_{2},-1}\left(t\right)\left(t\right),\;M_{x}\left(t\right)\;=\;\phi_{\alpha_{3},\beta_{3}}\left(t\right),$$

where the values of the parameters $\alpha_{j}$ and $\beta_{k}$ (k=1,2,3) can also be different.

## 5. Kicked GF Lorenz-Type Systems with Multi-Kernel Memory

From the examples given in the previous section, it is clear that despite the variety of memory functions, the parameters of memory functions are restricted by some intervals. For example, the parameter $\alpha_{j}$ of the function $M_{j}\left(t\right)=h_{\alpha_{j}}\left(t\right)$ must satisfy the condition $\alpha_{j}\in \left(0,1\right)$ so that $\left(M_{j}\left(t\right),K_{j}\left(t\right)\right)\in L_{1}$. To expand the range of parameter values, one can use the multi-kernel GFD and GFI proposed in works [43,44,45,69].

### 5.1. Kicked GF Lorenz Equations with Multi-Kernel GF Derivatives

Multi-kernel memory can be described by the convolutional product of the memory functions $M_{j}\left(t\right)$ for all j=1,…,m, which is denoted by

(84) $$M^{<1|m>}\left(t\right)\;=\;\left(M_{1}\;*\;\ldots \;*\;M_{m}\right)\left(t\right),$$

and $M^{<k|k>}\left(t\right)=M_{k}\left(t\right)$. For example,

(85) $$M^{<1|2>}\left(t\right)\;=\;\int_{0}^{t}M_{1}\left(t-s\right)\;M_{2}\left(s\right)\;ds,$$

(86) $$M^{<1|3>}\left(t\right)\;=\;\int_{0}^{t}M_{1}\left(t-s_{1}\right)\;\int_{0}^{s_{1}}M_{2}\left(s_{1}-s_{2}\right)\;M_{2}\left(s_{2}\right)\;ds_{2},$$

(87) $$M^{<1|m>}\left(t\right)\;=\;\int_{0}^{t}M_{1}\left(t-s_{1}\right)\;\int_{0}^{s_{1}}\;\ldots \;\int_{0}^{s_{m-2}}M_{m-1}\left(s_{m-2}-s_{m-1}\right)\;M_{m}\left(s_{m-1}\right)\;ds_{m-1}.$$

If $M_{j}=M\left(t\right)$ for all j=1,…,m, then $M^{<1|m>}\left(t\right)$ is denoted by the convolutional power $M^{*m}\left(t\right)$.

The multi-kernel GFD of the Caputo type is defined as

(88) $$D_{\left(K\right)}^{<1|m>,t}\left[s\right]\;f\left(s\right)\;:=\;D_{\left(K_{1}\right)}^{t,*}\left[t_{2}\right]\;\ldots \;D_{\left(K_{m}\right)}^{t_{m},*}\left[s\right]\;f\left(s\right),$$

where $\left(M_{j},\;K_{j}\right)\in L_{1}$ for all j=1,…,m and

(89) $$f\left(t\right)\;\in \;C_{-1}^{m}\left(0,\infty \right)\;:=\;\left\{f\left(t\right):\;f^{\left(m\right)}\left(t\right)\in C_{-1}\left(0,\infty \right)\right\}.$$

For m=1, the multi-kernel GFD is the GFD of the Caputo type

(90) $$D_{\left(K\right)}^{<j|j>,t}\left[s\right]\;f\left(s\right)\;:=\;D_{\left(K_{j}\right)}^{t,*}\left[s\right]\;f\left(s\right)\;=\;\int_{0}^{t}K_{j}\left(t-\left(s\right)\right)\;f^{\left(1\right)}\left(s\right)\;d\left(s\right),$$

where $f^{\left(1\right)}\left(x\right)=df\left(x\right)/dx$ and $f\left(t\right)\in C_{-1}^{1}\left(0,\infty \right)$.

Using Theorem 7 of the paper [69], one can derive Lorenz-type maps from the equations with multi-kernel GFD (88) and periodic kicks.

Let a kicked GF Lorenz-type system be described by the equation

(91) $$D_{\left(K\right)}^{<1|n>,t}\left[s\right]\;X\left(s\right)\;=\;A\;F_{L,\eta }\left(X\left(t\right)\right)\sum_{j=1}^{\infty }\delta \left(t/\tau -j\right),$$

where it is assumed that $\left(M_{j}\left(t\right),K_{j}\left(t\right)\right)\in L_{1}$ and $X\left(t\right)\in C_{-1}^{m}\left(0,\infty \right)$.

Let us denote the multi-kernel GFDs of the function X(t) as

(92) $$P_{k}^{*}\left(t\right)\;:=\;D_{\left(K\right)}^{<n-k+1|n>,t}\left[s\right]\;X\left(s\right)\;\left(k=1,\ldots ,n-1\right)$$

Then, Equation (91) gives

(93) $$D_{\left(K\right)}^{<1|n-k>,t}\left[s\right]\;P_{k}^{*}\left(s\right)\;=\;A\;F_{L,\eta }\left(X\left(t\right)\right)\sum_{j=1}^{\infty }\delta \left(t/\tau -j\right),$$

Equations (91) and (93) are analogous to the equations for coordinates and momenta in standard theory with two-dimensional phase space [1,2,3,4,5,6,24].

Using Theorem 7 of the paper [69], we get that Equation (91) for τm<t<τ(m+1) has the exact analytical solutions

$$X\left(s\right)\;=\;X\left(0\right)\;+\;\sum_{q=1}^{n-1}P_{q}^{*}\left(0\right)\;g_{<n-q+1|n>}\left(s\right)\;+\;$$

(94) $$A\;\tau \;\sum_{j=1}^{m}M^{<1|n>}\left(s-\tau j\right)\;F_{L,\eta }\left(X\left(\tau j\right)\right),$$

$$P_{k}^{*}\left(s\right)\;=\;P_{k}^{*}\left(0\right)\;+\;\sum_{q=k+1}^{n-1}P_{q}^{*}\left(0\right)\;g_{<n-q+1|n-k>}\left(s\right)\;+\;$$

(95) $$A\;\tau \;\sum_{j=1}^{m-1}M^{<1|n-k>}\left(s-\tau j\right)\;F_{L,\eta }\left(X\left(\tau j\right)\right),$$

where

(96) $$g_{<n-q+1|n-k>}\left(t\right)\;=\;\;\int_{0}^{t}M^{<n-q+1|n-k>}\left(s\right)\;ds.$$

The GF Lorenz-type discrete map for the function

(97) $$X_{j}\;=\;\lim_{\omega \to 0+}X\left(\tau j-\omega \right),\;\left(j=1,\ldots ,m+1\right),$$

is derived from solution (94) in the form

$$X_{m+1}\;-\;X_{m}\;=\;\sum_{q=1}^{n-1}X^{\left(q\right)}\left(0\right)\;\left(g_{<n-q+1|n>}\left(\tau \left(m+1\right)\right)\;-\;g_{<n-q+1|n>}\left(\tau m\right)\right)\;+\;$$

(98) $$A\;\tau \;M^{<1|n>}\left(\tau \right)\;F_{L}\left(X_{m}\right)\;+\;A\;\tau \;\sum_{j=1}^{m-1}L_{\left(M\right)}^{<1|n>}\left(\tau ,m-j\right)\;F_{L}\left(X_{j}\right)),$$

where

(99) $$L_{\left(M\right)}^{<1|n>}\left(\tau ,m-j\right)\;:=\;M^{<1|n>}\left(\tau \left(m-j+1\right)\right)\;-\;M^{<1|n-k>}\left(\tau \left(m-j\right)\right).$$

For functions (92), we define

(100) $$P_{k,j}^{*}\;:=\;\lim_{\omega \to 0+}P_{k}^{*}\left(\tau j-\omega \right),\;\left(j=1,\ldots ,m+1\right).$$

Then, solution (95) gives the discrete maps

$$P_{k,m+1}^{*}-P_{k,m}^{*}=\sum_{q=k+1}^{n-1}P_{q}^{*}\left(0\right)\;\left(g_{<n-q+1|n-k>}\left(\tau \left(m+1\right)\right)\;-\;g_{<n-q+1|n-k>}\left(\tau m\right)\right)\;+\;$$

(101) $$A\;\tau \;M^{<1|n-k>}\left(\tau \right)\;F_{L,\eta }\left(X_{m}\right)\;+\;A\;\tau \;\sum_{j=1}^{m-1}L_{\left(M\right)}^{<1|n-k>}\left(\tau ,m-j\right)\;F_{L,\eta }\left(X_{j}\right)),$$

where

(102) $$L_{\left(M\right)}^{<1|n-k>}\left(\tau ,m-j\right)\;:=\;M^{<1|n-k>}\left(\tau \left(m-j+1\right)\right)\;-\;M^{<1|n-k>}\left(\tau \left(m-j\right)\right),$$

and k=1,…,n−1.

### 5.2. Kicked GF Lorenz Equations with Multi-Kernel GF Operators

Multi-kernel memory can be also described by the operators

(103) $$D_{\left(K^{<1|m>}\right)}^{t,*}\left[s\right]\;f\left(s\right)\;:=\;I_{\left(K^{<1|m>}\right)}^{t}\left[s\right]\;f^{\left(m\right)}\left(s\right)\;=\;\int_{0}^{t}K^{<1|m>}\left(t-s\right)\;f^{\left(m\right)}\left(s\right)\;ds$$

which are proposed in [45,69]. For m=1, operator (103) is

(104) $$D_{\left(K^{<1|1>}\right)}^{t,*}\left[s\right]\;f\left(s\right)\;:=\;D_{\left(K_{1}\right)}^{t,*}\left[s\right]\;f\left(s\right).$$

Operator (103) can be interpreted as a multi-kernel GFD of the *m*-order, if $K^{<1|m>}\left(t\right)\;\in \;C_{-1,0}\left(0,\infty \right)$.

Using Theorem 9 of the paper [69], one can derive Lorenz-type maps from the equations with the operator $D_{\left(K^{<1|m>}\right)}^{t,*}\left[u\right]$ and periodic kicks.

Let a kicked GF Lorenz-type system be described by an equation with operator (104) as

(105) $$D_{\left(K^{<1|n>}\right)}^{t,*}\left[s\right]\;X\left(s\right)\;=\;A\;F_{L,\eta }\left(X\left(t\right)\right)\sum_{j=1}^{\infty }\delta \left(t/\tau -j\right)$$

where $\left(M_{j}\left(t\right),K_{j}\left(t\right)\right)\in L_{1}$ and $X\left(t\right)\in C_{-1}^{n}\left(0,\infty \right)$.

Then, using Theorem 9 of the paper [69], Equation (105) for τm<t<τ(m+1) has the exact analytical solution

(106) $$X\left(t\right)\;=\;\sum_{k=0}^{n-1}X^{\left(k\right)}\left(0\right)\;h_{k+1}\left(t\right)\;+\;A\;\tau \;\sum_{j=1}^{m}M^{<1|n>}\left(t-\tau j\right)\;F_{L}\left(X\left(\tau j\right)\right).$$

The GF Lorenz-type discrete map for the function

(107) $$X_{j}\;:=\;\lim_{\omega \to 0+}X\left(\tau j-\omega \right),\;\left(j=1,\ldots ,m+1\right),$$

is derived from solution (106) in the form

$$X_{m+1}\;-\;X_{m}\;=\;\sum_{q=0}^{n-1}X^{\left(q\right)}\left(0\right)\;\left(h_{q+1}\left(\tau \left(m+1\right)\right)\;-\;h_{q+1}\left(\tau m\right)\right)\;+\;$$

(108) $$A\;\tau \;M^{<1|n>}\left(\tau \right)\;F_{L,\eta }\left(X_{m}\right)\;+\;A\;\tau \;\sum_{j=1}^{m-1}L_{\left(M\right)}^{<1|n>}\left(\tau ,m-j\right)\;F_{L}\left(X_{j}\right),$$

where

(109) $$L_{\left(M\right)}^{<1|n>}\left(\tau ,m-j\right)\;:=\;M^{<1|n>}\left(\tau \left(m-j+1\right)\right)\;-\;M^{<1|n>}\left(\tau \left(m-j\right)\right).$$

For the derivatives $X^{\left(k\right)}\left(t\right)$ of integer order k>0, one can define the functions

(110) $$X_{j}^{\left(k\right)}\;,:=\;\lim_{\omega \to 0+}X^{\left(k\right)}\left(\tau j-\omega \right),\;\left(j=1,\ldots ,m+1\right).$$

Then, solution (106) gives the maps

$$X_{m+1}^{\left(k\right)}\;-\;X_{m}^{\left(k\right)}\;=\;\sum_{q=0}^{n-k-1}X^{\left(q+k\right)}\left(0\right)\;\left(h_{q+1}\left(\tau \left(m+1\right)\right)\;-\;h_{q+1}\left(\tau m\right)\right)\;+\;$$

(111) $$A\;\tau \;{\left(M^{<1|n>}\right)}^{\left(k\right)}\left(\tau \right)\;F_{L}\left(X_{m}\right)\;+\;A\;\tau \;\sum_{j=1}^{m-1}L_{k,j}^{<1|n>}\left(\tau ,m-j\right)\;F_{L}\left(X_{j}\right),$$

where

(112) $$L_{k,j}^{<1|n>}\left(\tau ,m-j\right)\;:=\;\left(D^{k}\;M^{<1|n>}\right)\left(\tau \left(m-j+1\right)\right)\;-\;\left(D^{k}M^{<1|n>}\right)\left(\tau \left(m-j\right)\right),$$

(113) $$\left(D^{k}\;M^{<1|n>}\right)\left(t\right)\;:=\;\frac{d^{k}M^{<1|n>}\left(t\right)}{dt^{k}},$$

and k=1,…,n−1.

### 5.3. Examples of Multi-Kernel Memory Functions

Let us give some examples of multi-kernel memory functions. To derive these functions by integration (87), one can use Table 9.1 in [17], p.173.

**Example** **1.** *For the memory functions*

(114) $$M_{1}\left(t\right)\;=\;h_{\mu }\left(t\right),\;M_{2}\left(t\right)\;=\;h_{\nu }\left(t\right),$$

*where μ,ν∈(0,1), we get*

(115) $$M^{<1|2>}\left(t\right)\;=\;(h_{\mu }\;*\;h_{\nu }\left(t\right)\;=\;h_{\mu +\nu }\left(t\right),$$

*where μ+ν∈(0,2) and*

(116) $$L_{\left(M\right)}^{<1|2>}\left(\tau ,m\right)\;=\;h_{\mu +\nu }\left(\tau \right)\;\left({\left(m+1\right)}^{\mu +\nu -1}\;-\;m^{\mu +\nu -1}\right).$$

*for positive integer values m.*

**Example** **2.** *For the memory functions*

(117) $$M_{1}\left(t\right)\;=\;h_{\mu ,\rho }\left(t\right),\;M_{2}\left(t\right)\;=\;h_{\nu ,\rho }\left(t\right),$$

*where μ,ν∈(0,1), we get*

(118) $$M^{<1|2>}\left(t\right)\;=\;\left(h_{\mu ,\rho }\;*\;h_{\nu ,\rho }\right)\left(t\right)\;=\;h_{\mu +\nu ,\rho }\left(t\right),$$

*and*

(119) $$L_{\left(M\right)}^{<1|2>}\left(\tau ,m\right)\;=\;h_{\mu +\nu ,m\rho }\left(\tau \right)\;\left({\left(m+1\right)}^{\mu +\nu -1}\;e^{-\rho \tau }\;-\;m^{\mu +\nu -1}\right).$$

*If the memory functions are*

(120) $$M_{j}\left(t\right)\;=\;M_{1}\left(t\right)\;=\;h_{\mu ,\rho }\left(t\right),\;M_{n+j}\left(t\right)\;=\;M_{2}\left(t\right)\;=\;h_{\nu ,\rho }\left(t\right),$$

*for all j=1,…,n, then*

(121) $$M^{<1|2n>}\left(t\right)\;=\;{\left(M^{<1|2>}\right)}^{*,n}\left(t\right)\;=\;h_{k(\mu +\nu ),\rho }\left(t\right),$$

*where n(μ+ν)∈(0,2n), and*

(122) $$L_{\left(M\right)}^{<1|2k>}\left(\tau ,m\right)\;=\;h_{n(\mu +\nu ),m\rho }\left(\tau \right)\;\left({\left(m+1\right)}^{n(\mu +\nu )-1}e^{-\rho \tau }\;-\;m^{n(\mu +\nu )-1}\right)$$

*with positive integer values m and n(μ+ν)∈(0,2n).*

**Example** **3.** *Let us take*

(123) $$M_{1}\left(t\right)\;=\;e_{\alpha ,\beta ,-1}\left(t\right),\;M_{2}\left(t\right)\;=\;h_{\mu }\left(t\right),$$

*where μ∈(0,1) and 0<α≤β<1. Then*

(124) $$M^{<1|2>}\left(t\right)\;=\;\left(h_{\mu }\;*\;e_{\alpha ,\beta ,-1}\right)\left(t\right)\;=\;e_{\alpha ,\beta +\mu ,-1}\left(t\right),$$

*and*

(125) $$L_{\left(M\right)}^{<1|2>}\left(\tau ,m\right)\;=\;e_{\alpha ,\beta +\mu ,-1}\left(\left(m+1\right)\tau \right)\;-\;e_{\alpha ,\beta +\mu ,-1}\left(m\tau \right),$$

*where β+μ∈(0,2).*

**Example** **4.** *For the functions*

(126) $$M_{j}\left(t\right)\;=\;h_{\mu_{j}}\left(t\right),\;\left(j\;=\;1,\;\ldots ,\;n\right),$$

*where $\mu_{j}\;\in \;\left(0,1\right)$, we get*

(127) $$M^{<1|n>}\left(t\right)\;=\;h_{\mu }\left(t\right),\;\mu \;:=\;\sum_{j=1}^{n}\mu_{j}\in \left(0,n\right),$$

*and*

(128) $$L_{\left(M\right)}^{<1|n>}\left(\tau ,m\right)\;=\;h_{\mu }\left(\tau \right)\;\left({\left(m+1\right)}^{\mu -1}\;-\;m^{\mu -1}\right),$$

*where μ∈(0,n).*

**Example** **5.** *For the functions*

(129) $$M_{j}\left(t\right)\;=\;h_{\mu_{j},\rho }\left(t\right),\;\left(j=1,\ldots ,n\right),$$

*where $\mu_{j}\in \left(0,1\right)$ and ρ>0, we get*

(130) $$M^{<1|m>}\left(t\right)\;=\;h_{\mu ,\rho }\left(t\right),\;\mu \;:=\;\sum_{j=1}^{n}\mu_{j}\in \left(0,n\right),$$

*and*

(131) $$L_{\left(M\right)}^{<1|n>}\left(\tau ,m\right)\;=\;h_{\mu ,m\rho }\left(\tau \right)\;\left({\left(m+1\right)}^{\mu -1}\;e^{-\rho \tau }\;-\;m^{\mu -1}\right),$$

*where μ∈(0,n).*

**Example** **6.** *For the memory functions*

(132) $$M_{1}\left(t\right)\;=\;e_{\alpha ,\beta ,-1}\left(t\right),\;M_{j}\left(t\right)\;=\;h_{\mu_{j}}\left(t\right)$$

*where j=2,…,n, $\mu_{j}\in \left(0,1\right)$ and 0<α≤β<1, we derive*

(133) $$M^{<1|m>}\left(t\right)\;=\;\left(h_{\mu }\;*\;e_{\alpha ,\beta ,-1}\right)\left(t\right)\;=\;e_{\alpha ,\beta +\mu ,-1}\left(t\right),$$

*where*

(134) $$\mu \;:=\;\sum_{j=2}^{n}\mu_{j}\in \left(0,n-1\right),$$

*and*

(135) $$L_{\left(M\right)}^{<1|n>}\left(\tau ,m\right)\;=\;e_{\alpha ,\beta +\mu ,-1}\left(\left(m+1\right)\tau \right)\;-\;e_{\alpha ,\beta +\mu ,-1}\left(m\tau \right),$$

*where β+μ∈(0,n).*

## 6. Conclusions

In this work, the generalization of Lorenz-type systems by using GFC and periodical kicks is proposed. GFDs allow us use the general form of memory functions as the operator kernels to describe nonlinear dynamics with memory. The exact analytical solutions of nonlinear equations with GFDs are derived in the general case for the wide class of nonlinearity and memory functions. Using the exact solutions, we obtain discrete maps with memory that describe kicked GF Lorenz-type systems with general forms of memory and nonlocality. The proposed maps describe the exact solution of nonlinear equations with GFDs at discrete time points as the function of all past discrete moments of time. It should be emphasized that the proposed nonlinear discrete maps with memory are derived from kicked GF Lorenz-type equations with GFDs without any approximations. Let us emphasize the unusualness and importance of the proposed results: exact analytical solutions were derived for dissipative chaotic systems of the Lorentz type. As a rule, the fractional differential equations of Lorenz-type systems do not have exact analytical solutions.

It can be assumed that new types of chaotic behavior and new types of attractors, repellers, and limit cycles can be demonstrated in the proposed DMMs obtained from nonlinear equations with FDs in this paper as a generalization of the Edelman results. This is an important and very interesting direction of research, namely, the search for new types of chaotic behavior and a new type of attractors in dynamic maps with memory, which are exact solutions of equations with FDs. To date, new types of chaotic behavior have been described for one-dimensional mappings in the first papers [60,72,73] and then in the works [74,75,76,77,78]. The computer simulations for multi-dimensional discrete maps with memory are open questions at the present time and require new researches to make great discoveries in the future.

Let us note that fractional calculus is actively developing and has a lot of applications (for example, see [117,118,119,120,121]). The proposed approach to obtaining the exact solutions of fractional differential equations and discrete maps with memory can be applied to multi-kernel generalizations of other types of general fractional derivatives. For example, it can be applied to generalizations of Hilfer derivatives [67,122,123] and their single-kernel general fractional analogs [124].

## Data Availability

No new data were created or analyzed in this study. Data sharing is not applicable to this article.
